# Mechanism of NURP1 in temozolomide resistance in hypoxia-treated glioma cells via the KDM3A/TFEB axis

**DOI:** 10.32604/or.2023.028724

**Published:** 2023-05-24

**Authors:** TAO LI, XIN FU, JIE WANG, WEI SHANG, XIAOTONG WANG, LINYUN ZHANG, JUN LI

**Affiliations:** Department of Neurosurgery, First Affiliated Hospital of Dalian Medical University, Dalian, 116011, China

**Keywords:** NUPR1, Autophagy, TMZ resistance, Glioma, KDM3A

## Abstract

Temozolomide (TMZ) resistance is a major obstacle in glioma treatment. Nuclear protein-1 (NUPR1) is a regulator of glioma progression. This study investigated the mechanism of NUPR1 in TMZ resistance in hypoxia-treated glioma cells and its mechanism in modulating autophagy. We treated TMZ-resistant cells U251-TMZ and T98G-TMZ to normoxia or hypoxia and silenced NUPR1 in hypoxia-treated U251-TMZ and T98G-TMZ cells to assess cell viability, proliferation, apoptosis, LC3-II/LC3-I and p62 expressions, and autophagic flux under different concentrations of TMZ. We found that hypoxia upregulated NUPR1 expression and autophagy while NUPR1 silencing suppressed hypoxia-induced TMZ resistance and autophagy in glioma cells. We also investigated the interaction between NUPR1 and lysine demethylase 3A (KDM3A), as well as the enrichments of KDM3A and H3 lysine 9 dimethylation (H3K9me2) in the transcription factor EB (TFEB) promoter region. Our results suggest that hypoxia-induced NUPR1 promotes TFEB transcription by binding to KDM3A and reducing H3K9me2 levels, thereby augmenting glioma cell autophagy and TMZ resistance. Moreover, the overexpression of KDM3A or TFEB promoted glioma cell autophagy. In a xenograft tumor model, silencing NUPR1 suppressed TMZ resistance in glioma cells *in vivo*. Overall, our findings highlight a mechanism by which NUPR1 enhances glioma cell autophagy and TMZ resistance via the KDM3A/TFEB axis.

## Introduction

Glioma, including astrocytoma, glioblastoma (GBM), and ependymoma, are primary tumors originating from the neuroepithelium, accounting for approximately 70% of malignant intracranial tumors [1]. According to the authoritative classification, gliomas are divided into 4 grades, with grades I and II considered as low-grade glioma and grades III and IV as high-grade ones, with the latter having a poor prognosis and survivorship [2]. GBM is classified as grade IV, and the current therapeutic methods of GBM include surgery, radio-chemotherapy, and temozolomide (TMZ) [3]. TMZ, a DNA alkylating agent, induces cell apoptosis through the addition of methyl groups on urine and pyrimidine of DNA [4]. Unfortunately, over 50% of GBM patients develop resistance to TMZ, leading to ineffective clinical treatment with TMZ-based therapies [5]. Hypoxia, a representative mark of the tumor microenvironment (TME), is prevalent in multiple tumors and fosters these tumors with figurability and heterogeneity, resulting in a more metastatic and invasive phenotype [6]. Existing studies documented that hypoxia augments therapeutic resistance in glioma [7,8]. Additionally, autophagy, a highly-regulated self-degradation process, achieves the degradation of intracellular elements through autolysosomes [9]. Interestingly, autophagy exerts dichotomous functions in cancer by suppressing tumor initiation or facilitating tumor development [10], and hypoxia induced autophagy in tumors develops therapeutic resistance in GBM [11]. Therefore, in this study, we strived to analyze the specific roles of hypoxia and hypoxia-induced autophagy in TMZ resistance in glioma cells.

Nuclear protein1 (NUPR1/Com1/p8) is a multifunctional nuclear protein located on chromosome 16p11.2, could respond to multiple stress stimuli [12]. NUPR1 has demonstrated functionality in the tumorigenesis and progression of multiple cancers can confer resistance to relative chemotherapies, such as sorafenib, docetaxel, and tamoxifen [13–16]. Additionally, NUPR1 has been implicated in glioma development by affecting cell cycle arrest [17]. Furthermore, NUPR1 can promote cancer cell proliferation and migration by activating autophagy [18,19]. However, the specific role of NUPR1 on TMZ resistance in glioma cells through autophagy remains unclear.

Lysine demethylase 3A (KDM3A) is a specific demethylase for H3 lysine 9 dimethylation (H3K9me2) and has been implicated in cancer development and chemoresistance, making it a promising therapeutic target [20,21]. A existing study has highlighted the involvement of KDM3A in the development of GBM [22]. Additionally, KDM3A has been shown to induce autophagy under conditions of nutrient deprivation [23]. We have also identified a binding relation between KDM3A and NUPR1. Nevertheless, the role of KDM3A in glioma from perspective of resistance to TMZ remains elusive. Notably, a transcription factor EB (TFEB), a vital regulator for lysosome biogenesis has elicited a regulating role in autophagy [24,25]. Fang et al. reported that TFEB overexpression activates autophagy and enhances chemotherapy resistance [26]. Moreover, our investigation revealed an association between the binding of NUPR1 to KDM3A and TFEB transcription. Therefore, we sought to validate the potential association and functionality of NURP1/KDM3A/TFEB in the context of TMZ resistance in glioma cells.

In conclusion, the present study aims to investigate the potential regulatory role of NURP1 may regulate the KDM3A/TFEB axis to modulate autophagy under hypoxia, and this process may affect TMZ resistance in glioma cells. By investigating the potential association and roles of NURP1/KDM3A/TFEB, we aim to provide new insights into the molecular route of TMZ resistance in glioma cells.

## Materials and Methods

### Construction of TMZ-resistant cells

GBM cell lines U251 and T98G (ATCC, VA, USA) and were cultured in Dulbecco’s modified Eagle’s medium (DMEM; Invitrogen, Carlsbad, CA, USA) supplemented by a combination of 10% fetal bovine serum, and 100 U/ml penicillium, and 100 μg/ml streptomycin (1% PenStrep) in a moist condition at 37°C with 5% CO_2_. For establishment of TMZ-resistant cells U251-TMZ and T98G-TMZ, the parental U251 and T98G cells underwent exposure to 10–100 μM TMZ for 6 months until the appearance of a stable TMZ-resistant phenotype. The aforementioned cell populations of resistant U251-TMZ and T98G-TMZ were immersed in 96-well plates as monoplasts for incubation with 150 μM TMZ [bought from Selleck Chemicals corporation (Houston, TX, USA) and pre-dissolved in dimethylsulfoxide (DMSO, Invitrogen, Carlsbad, CA, USA)] for 3 weeks to harvest TMZ-resistant cells U251-TMZ and T98G-TMZ. The three-gas incubator (5% CO_2_, 93% N_2_, and 2% O_2_; YCP-50S, BLOOMAGE BIOTECH Co., Ltd., Changsha, Hunan, China) was used for preparation of cell hypoxia culture at 37°C for 24 h [27,28].

### Cell transfection and treatment

The coding DNA sequences (CDS) of KDM3A and TFEB were inserted into the pcDNA3.1 vectors (Thermo Fisher Scientific, Waltham, MA, USA). Plasmids pcDNA-KDM3A (oe-KDM3A), pcDNA-TFEB (oe-TFEB), and the corresponding empty vector (oe-NC), as well as siRNA sequences of NUPR1 (si-NUPR1) and the corresponding control (si-NC) were all supplied by GenePharma (Shanghai, China). To induce autophagy, DMSO-soluble rapamycin was obtained from Sigma (St. Louis, MO, USA) [29], and a dose of 0.5 μM was added to treat cells U251-TMZ or T98G-TMZ cells for 1 h. Thereafter, according to standard procedures in the instructions, the plasmids were transfected into U251-TMZ or T98G-TMZ cells using Lipofectamine 3000 (Thermo Fisher Scientific). After 48 h of transfection, hypoxia treatment, TMZ treatment, and subsequent experiments were performed. The siRNA sequences are shown in Table 1.

**Table 1:** si-RNAs sequence

siRNAs	Sequence
si-NUPR1#1	5′-CCTCTCATCATGCCTATGCCCACTT-3′
si-NUPR1#2	5′-TCTCATCATGCCTATGCCCACTTCA-3′
si-NC	5′-CATCACAGACAGATTATGATGTGAT-3′

### Cell counting kit-8 (CCK-8) assay

The cells were grown in 96-well plates at a density of 5 × 10^3^ cells/well and treated with variable concentrations of TMZ with (0, 100, 200, 300, 400, and 500 μM) at 37°C for 24 h [30]. Next, the incorporation of 10 µL of CCK-8 reagent (Beyotime, Shanghai, China) into each well was accompanied by 2 h incubation at 37°C with 5% CO_2_. The absorbance was examined at an excitation wavelength of 450 nm using a microplate reader (Bio-Rad 680, Bio-Rad, Hercules, CA, USA). The IC_50_ values, indicative of TMZ concentrations that inhibits 50% cell activity, with higher IC_50_ values indicating higher resistance potential. This cell experiment was performed in triplicate. TMZ concentrations corresponding to the IC_50_ value of the TMZ-resistant cells were utilized for all subsequent experiments.

### Flow cytometry (FCM)

Apoptosis was assessed by means of double-staining dependent on Annexin V FITC/propidium iodide (PI) (Sigma, St. Louis, MO, USA). The cell density was adjusted to a density of 1 × 10^6^ cells/mL and fixed overnight with 70% pre-cooling ethanol at 4°C. Next, a 100 μL cell suspension (≥10^6^ cells/mL) was transferred to 200 μL binding buffer, followed by 15 min staining with 10 μL Annexin V-FITC and 5 μL PI at ambient temperature in the dark. Following incorporation of 300 μL binding buffer, apoptosis was analysed by flow cytometry (MoFloAstrios EQ, Beckman Coulter., Inc., Fullerton, CA, USA) with excitation wavelength at 488 nm, with 2 × 10^4^ cells analysed each time. This cell experiment was performed in triplicate.

### Quantitative polymerase chain reaction (qPCR)

The total RNA content was isolated by employing RNeasy Mini kits (Qiagen, Valencia, CA, USA) and converted into cDNA with application of the RR047A reverse transcription kit (Takara, Tokyo, Japan). qRT-PCR analysis ran with the help of the DRR081 SYBR^®^ Premix Ex Taq^TM^ II kit (Takara) and ABI 7500 Real-time fluorescent quantitative PCR instrument (ABI, Foster City, CA, USA). The two steep method was employed for PCR amplification: pre-denaturation at 95°C for 30 s; accompanied by 40 cycles of PCR reaction at 95°C for 5 s and at 60°C for 30 s, with the setting of at least 3 duplicate wells for each specimen. The primers were synthesized by Sangon Biotech Sangon Biotech (Shanghai, China) and listed in Table 2. Ct values of each well were recorded; where β-actin served as the internal reference; the relative expression was calculated based on the 2^−ΔΔCt^ method; ΔΔCt = (averaged Ct value of target gene in experimental group − averaged Ct value of housekeeping gene in experimental group) − (averaged Ct value of target gene in control group − averaged Ct value of housekeeping gene in control group).

**Table 2:** qPCR primers

	Forward primer (5′-3′)	Reverse primer (5′-3′)
NUPR1	GCACGAGAGGAAACTGGTGA	GTCCCGTCTCTATTGCTGGGGG
TFEB	ACCTGTCCGAGACCTATGGG	CGTCCAGACGCATAATGTTGTC
β-actin	AAGGACTCCTATAGTGGGTGACGA	ATCTTCTCCATGTCGTCCCAGTTG

### Western blot

The extraction of the total protein content from tissues or cells was conducted using enhanced RIPA buffer containing protease inhibitor (Boster Biological Technology Co., Ltd., Wuhan, Hubei, China), with bicinchoninic acid protein kits for quantification. The protein was subjected to 10% sodium dodecyl sulfate-polyacrylamide gel electrophoresis and transferred to a polyvinylidene fluoride membrane (Thermo Fisher Scientific, Waltham, MA, USA). After 2 h pretreatment with 5% bovine serum albumin to prevent non-specific binding, membranes underwent overnight incubation at 4°C with specific primary antibody diluted in a blocking buffer: rabbit-anti NUPR1 antibody (at a dilution ratio 1:1000, PA1-4177, Invitrogen), β-actin (at a dilution ratio 1:2000, ab8227, Abcam, Cambridge, UK), LC3 I/II (at a dilution ratio 1:3000, ab51520, Abcam), p62 (at a dilution ratio 1:10000, ab109012, Abcam), KDM3A (at a dilution ratio 1:2000, ab80598, Abcam), H3K9me2 (at a dilution ratio 1:1000, ab176882, Abcam), and TFEB (at a dilution ratio 1:1000, ab267351, Abcam). After rinsing, the membrane was incubated with horseradish peroxidase (HRP)-labeled goat anti-rabbit secondary antibody IgG (at a dilution ratio 1:2000, ab205718, Abcam) for 1 h at room temperature. The proteins bands were visualised using an enhanced chemiluminescence reagent (EMD Millipore, Billerica, MA, USA) and quantified using the Image Pro Plus 6.0 (Media Cybernetics, San Diego, CA, USA). β-actin severed as the internal control to calculate the relative expression of each protein. The experiment was conducted 3 times independently.

### Colony formation assay

The transfected U251-TMZ or T98G-TMZ cells were digested using trypsin, grown in 6-well plates (1 × 10^3^ cells/well), and cultured for 2 to 3 weeks. After formation of visible colonies, they underwent 10 min fixation with 4% paraformaldehyde for 10 and 10 min staining with 0.1% crystal violet (Sigma-Aldrich, St. Louis, MO, USA) at room temperature. The stained colonies (>50 cells) were counted under a DMM-300D microscope (Magnification × 40; Caikon, Shanghai, China). This cell experiment was performed in triplicate.

### mRFP-GFP-LC3 double fluorescent labeling

The transfection with mRFP-GFP-LC3 adenovirus vectors (HanBio Technology, Shanghai, China) was conducted to visualise autophagosomes and autolysosomes in U251-TMZ or T98G-TMZ cells. After fixation with 4% paraformaldehyde, the cells were examined under a Zeiss 780 upright confocal microscope (Carl Zeiss, Jena, Germany). Quantitative analysis was performed on approximately 100 cells from 10 to 20 randomly fields (×630). The GFP-LC3 as well as mRFP-LC3 dot number in per cell was counted via Image Pro Plus 6.0 (Media Cybernetics). The number of autophagosomes = GFP-LC3 dots; the number of autolysosome = mRFP-LC3 dots − GFP-LC3 dots; the autophagic flux = the number of autolysosome/the total number of mRFP dots.

### Co-immunoprecipitation (Co-IP)

U251-TMZ or T98G-TMZ cells cultured in normoxic or hypoxic conditions were transfected with GFP-tagged-NUPR1 (GFP-NUPR1). For cells in hypoxic conditions, GFP-NUPR1 and si-NC or si-NUPR1 were transfected for 24 h. The cells of each group were lysed on ice by adding HEPES based lysis buffer supplemented with proteases inhibitor cocktail (at a dilution ratio of 1:200; Sigma P8340) and subsequently centrifuged at 1,400 × g for 10 min at 4°C to isolate proteins in the supernatant for protein concentration determination. A Co-IP assay was conducted using GFP trap^®^ beads (ChromoTek, Munich, Germany) following standard procedures in instructions. The immunoprecipitate was rinsed 3 times with lysis buffer, followed by a rinse with using phosphate-buffered saline (PBS). Finally, antibodies of KDM3A (at a dilution ratio of 1:2000, ab80598, Abcam) and GFP (at a dilution ratio of 1:2000, ab6556, Abcam) were used for Western blot assay.

### Chromatin immunoprecipitation (Ch-IP)

To generate DNA-protein cross-links, the cells underwent 10 min fixation with formaldehyde. The ultrasonic fragmentation was then performed using an apparatus set to perform 10 s each time with an interval of 10 s and a circulation of 15 times to break chromatin into fragments. Next, chromatin fragments were incubated overnight at 4°C with antibody IgG (at a dilution ratio of 1:1000, ab172730, Abcam), target protein-specific antibody KDM3A (2 µg/mL, ab80598, Abcam), and H3K9me2 (2 µg/mL, ab176882, Abcam). Protein Agarose/Sepharose (Thermo Fisher Scientific, Waltham, MA, USA) was used to precipitate the DNA-protein complex, after which centrifugation ran at 12,000 × g for 5 min and the supernatant was removed. The non-specific complex was washed accompanied by overnight de-crosslink at 65°C. DNA fragments were recovered using phenol/chloroform extraction and purification. The binding of KDM3A and H3K9me2 was determined by qRT-PCR with TFEB-specific primers: 5′-GAAGGAGGAGAGAGAGGAAGAA-3′ (Sense), 5′-CTGGCGAAGTACAGGGTAAA-3′ (AntiSense).

### Animal models

A total of 24 BALB/c female nude mice with age of 4–6 weeks and weigh of 18–25 g (Shanghai SLAC Laboratory Animal Co., Ltd., China) were housed for one week under constant temperature (25°C–27°C) and humidity to adapt to their new environment. The animal care procedures and animal experiments were ratified by the Laboratory Animal Ethics Committee of First Affiliated Hospital of Dalian Medical University and complied with the *Guidelines for the Care and Use of Laboratory Animals* of the National Institutes of Health (NIH) and other relevant national regulations. The lentiviruses LV-sh-NC and LV-sh-NUPR1 were obtained from GenePharma. The mice were randomly allocated into two groups, LV-sh-NC group and LV-sh-NUPR1 group, with 12 mice per group. The lentivirus (multiplicity of infection = 10) was used to infect U251-TMZ cells when the cell growth rate reached 80% to 90% with the addition of 10 mg/mL polybrene [31]. After detachment and centrifugation, the cells were rinsed by PBS for 2 to 3 times, resuspended, and finally counted. Adjusted to cell density of 1 × 10^7^ cells/mL, 20 μL of the cell suspension was subcutaneously injected into the dorsum of each mouse to establish xenograft models. When the tumor volume reached 0.1 cm^3^, the mice were orally administered TMZ treatment (20 mg/kg/day; for 5 days a week; 3 periods) [28]. With vernier caliper to assess the tumor size every 5 days from the first treatment TMZ, the tumor volume was measured according to the following formula: tumor volume = (a × b^2^)/2; a: tumor’s longest diameter, b: tumor’s shortest diameter. Tumor growth curve was plotted according to tumor volume changes. After 25 days, with intraperitoneal injection of an overdose of pentobarbital sodium (100 mg/kg) for animal euthanasia, subcutaneous xenograft tumors were dissected and weighed [32]. Six tumors from each group were used for TdT-mediated dUTP Nick-End Labeling (TUNEL) staining and the remaining six tumors were homogenized, and the protein expression was examined by Western blot.

### TUNEL staining

To assess the level of apoptosis in the tumors, TUNEL staining (Thermo Fisher Scientific) was performed. Briefly, tumor samples were embedded in paraffin, sliced into 5 μm sections, which were then dewaxed, rehydrated and treated with proteinase K working solution for 30 min and subsequently with treated with H_2_O_2_ for 10 min at room temperature after a rinse with PBS. After another rinse with PBS, the slices were then treated with TUNEL reaction liquid at 37°C for 1 h in a dark humidified chamber. The sections were then washed and treated with HRP-labeled streptavidin and diaminobenzidine (DAB) for color development, with a microscope (Olympus, Tokyo, Japan) for observation.

### Statistical analysis

SPSS21.0 statistical software (IBM Corp., Armonk, NY, USA) and GraphPad Prism 9.0 statistical software (GraphPad Software Inc., San Diego, CA, USA) was utilized for implementation of statistical analysis and mapping of data. The measurement data were shown as mean ± standard deviation; tests of normality and homogeneity of variance were conducted initially to validate data conformation to normal distribution and homogeneity of variance; data between two groups were examined based on the *t*-test; one-way ANOVA or two-way ANOVA was used for comparisons among multiple groups; Tukey’s multiple comparison test or Tukey’s Honestly-Significant-Difference test was used for post-hoc test; The *p* value was obtained from the two-sided test. In all statistical references, a value of *p* < 0.05 denoted a statistically significant difference.

## Results

### Silencing NUPR1 repressed TMZ resistance in hypoxia-induced malignant glioma cells

**Figure 1:**
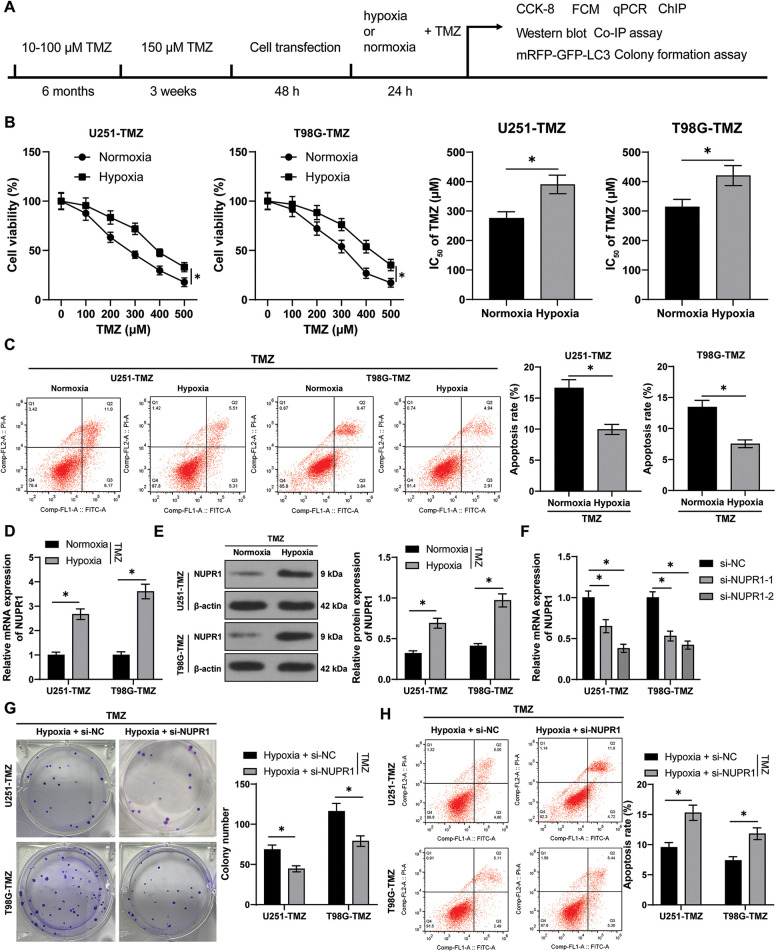
Silencing NUPR1 repressed hypoxia-induced TMZ resistance in glioma cells. U251-TMZ and T98G-TMZ cells were remained in normoxia and hypoxia conditions for 24 h, respectively. (A) Flow diagram of cell experiments; (B) CCK-8 assay was used to determine the cell activity under different concentrations of TMZ (0, 100, 200, 300, 400, and 500 μM) to acquire IC_50_ values. TMZ concentrations corresponding to IC_50_ values were used to treat U251-TMZ and T98G-TMZ cells, respectively. (C) FCM assay was conducted to examine apoptosis; (D and E) qPCR and Western blot were performed to examine NUPR1 expression; (F) Plasmids si-NC, si-NUPR1-1, and si-NUPR1-2 were transfected to hypoxia-treated U251-TMZ and T98G-TMZ cells, and qPCR was used to examine NUPR1 expression; (G) Colony formation assay was conducted to verify cell proliferation; (H) FCM was conducted to detect apoptosis. The cell experiment was conducted 3 times independently; the data were expressed as mean ± standard deviation; data in panels B (bar charts) and C were verified using *t*-test; data in panels B (line charts) and D-H were analyzed using two-way ANOVA, followed by Tukey’s Honestly-Significant-Difference test; *p < 0.05.

The experimental design (Fig. 1A) involved the construction of TMZ-resistant cells U251-TMZ and T98G-TMZ, followed by culture under normoxia and hypoxia conditions for 24 h, respectively. The cells were stimulated with different concentrations of TMZ (0, 100, 200, 300, 400, and 500 μM), the IC_50_ value of cells in the hypoxia group was significantly higher relative to the normoxia group (*p* < 0.05, Fig. 1B). The TMZ concentrations were in strict accordance with the IC_50_ values of these resistant cells (U251-TMZ: IC_50_ = 390.37; μM; T98G-TMZ: IC_50_ = 420.52 μM). Moreover, apoptosis was significantly reduced in the hypoxia group (*p* < 0.05, Fig. 1C) relative to the normoxia group. These findings indicate that hypoxia could increase the IC_50_ values of TMZ, reduce apoptosis, and promote TMZ resistance in glioma cells.

The hypoxia-treated cells exhibited high expression of NURP1 as revealed by qPCR and Western blot assay (*p* < 0.05, Figs. 1D and 1E). Subsequently, hypoxic U251-TMZ and T98G-TMZ cells were transfected with plasmids si-NC, si-NUPR1-1, and si-NUPR1-2. Among them, si-NUPR1-2 (uniformly named as si-NUPR1) with a superior silencing effect was selected for subsequent experimentation (*p* < 0.05, Fig. 1F). The results showed that si-NUPR1 could suppress TMZ-induced cell proliferation (*p* < 0.05, Fig. 1G) and accelerate apoptosis (*p* < 0.05, Fig. 1H). Collectively, these findings suggest that silencing NUPR1 could limit TMZ resistance in hypoxia-induced glioma cells.

### Silencing NUPR1 repressed hypoxia-induced TMZ resistance via limiting cell autophagy

**Figure 2:**
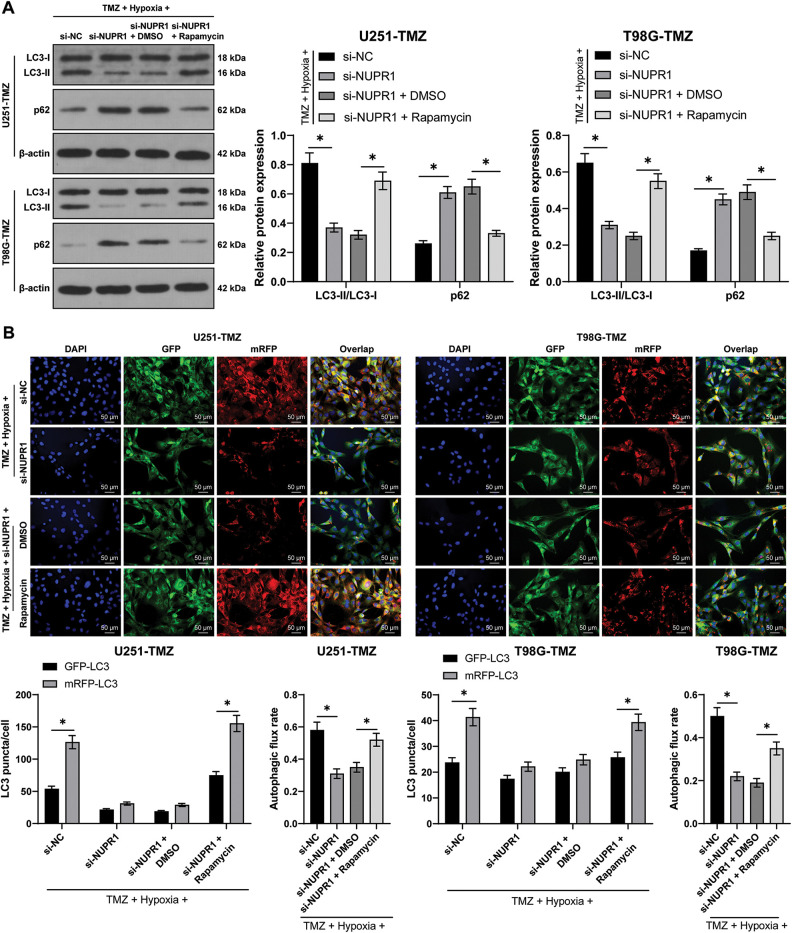

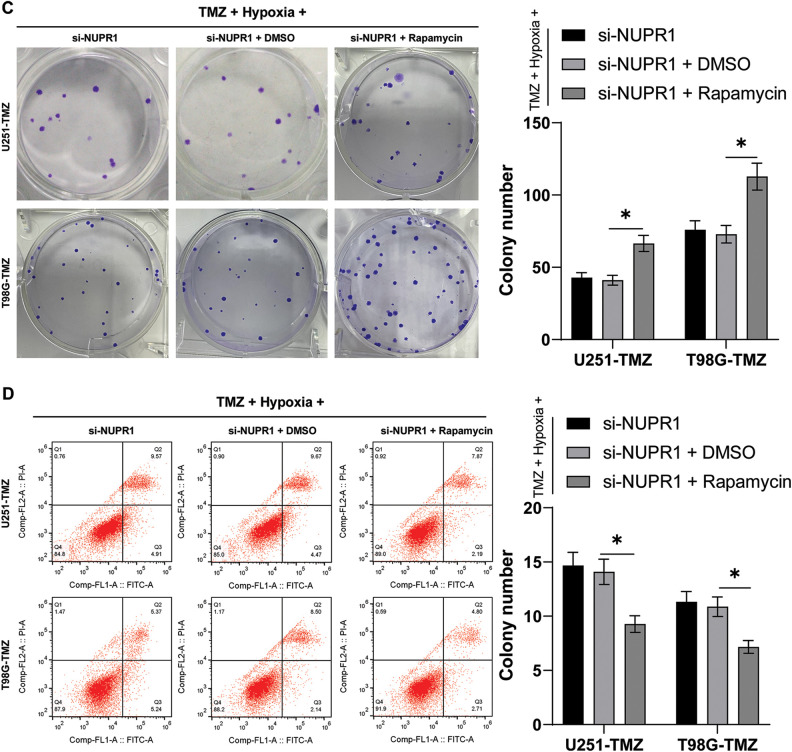
Silencing NUPR1 repressed hypoxia-induced TMZ resistance via limiting cell autophagy. Firstly, NUPR1 expression was suppressed in hypoxia-treated U251-TMZ and T98G-TMZ cells. TMZ concentrations corresponding to IC50 values were used to treat U251-TMZ and T98G-TMZ cells, followed by rapamycin treatment or without rapamycin treatment. (A) Western blot was performed to identify the expressions of LC3-II/LC3-I and p62; (B) mRFP-GFP-LC3 dual-fluorescence labeling was adopted to verify autophagy flux and calculate autophagy flux rate; (C) Colony formation assay was conducted to detect cell proliferation; (D) FCM was conducted to examine apoptosis. The cell experiment was conducted 3 times independently; the data were expressed as mean ± standard deviation; data in panel B (the second and fourth bar charts) were analyzed using one-way ANOVA, followed by Tukey’s multiple comparison test, and data in panels A, B (the first and third bar charts), C, and D were analyzed using two-way ANOVA, followed by Tukey’s Honestly-Significant-Difference test; _p < 0.05.

Hypoxia/TMZ-treated U251-TMZ and T98G-TMZ cells were transfected with plasmid si-NC or si-NUPR1. The results revealed that silencing NUPR1 decreased LC3-II/LC3-I expression and increased p62 expression in U251-TMZ and T98G-TMZ cells, whereas rapamycin (autophagy agonist) treatment partially reversed the effects of si-NUPR1 (*p* < 0.05, Fig. 2A). Additionally, mRFP-GFP-LC3 dual-fluorescence labeling revealed that hypoxia-induced autophagy flux was suppressed by si-NUPR1 but promoted after rapamycin treatment (*p* < 0.05, Fig. 2B), indicating that silencing NUPR1 could suppress hypoxia-induced autophagy. Moreover, rapamycin treatment facilitated cell proliferation (*p* < 0.05, Fig. 2C) and limited apoptosis (*p* < 0.05, Fig. 2D). The aforementioned findings suggest that silencing NUPR1 suppressed cell autophagy to repress hypoxia-induced TMZ resistance.

### NURP1 declined H3K9me2 level in glioma cells via binding to KDM3A

**Figure 3:**
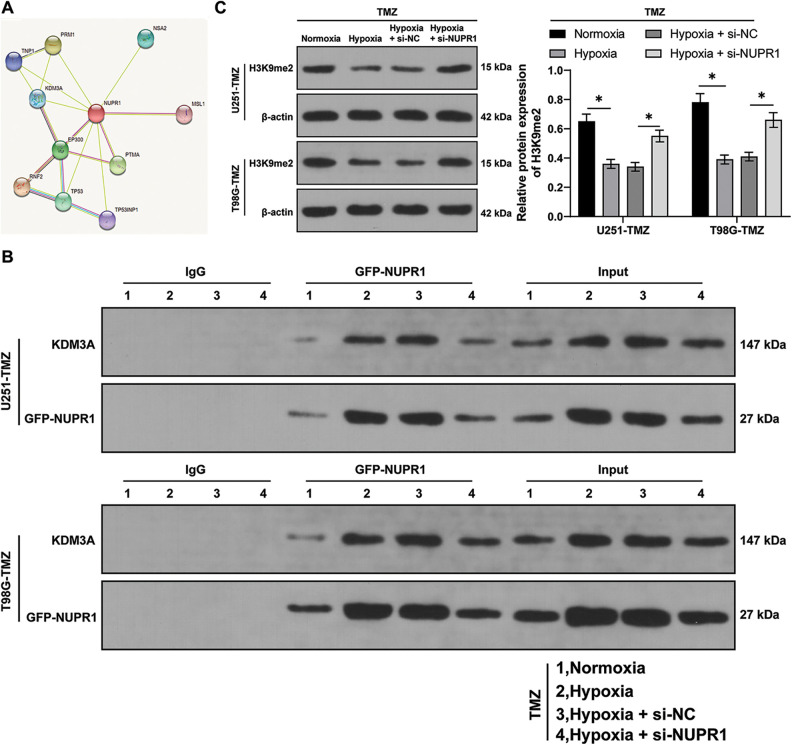
NURP1 declined H3K9me2 level in glioma cells via binding to KDM3A. (A) STRING database (https://cn.string-db.org/cgi/input.pl) was adopted to predict the binding relation between NUPR1 and KDM3A. NUPR1 expression was suppressed in hypoxia-treated U251-TMZ and T98G-TMZ cells. (B) Co-IP assay for GFP-NUPR1 was conducted to validate the binding relation between NUPR1 and KDM3A. (C) Western blot was performed to examine H3K9me2 expression. The cell experiment was conducted 3 times independently; the data were expressed as mean ± standard deviation; data in panel C were analyzed using two-way ANOVA, followed by Tukey’s Honestly-Significant-Difference test; *p < 0.05.

Firstly, the STRING database (https://cn.string-db.org/) was utilized to predict the relationship between NUPR1 and KDM3A (Fig. 3A). Moreover, an existing study reported that KDM3A can reduce the H3K9me2 enrichment in the promoter region of target gene promoter region by binding to them and transcriptionally activating the expressions of autophagy-related genes [23]. Afterwards, the NUPR1 expression in TMZ-treated normoxic or hypoxic U251-TMZ and T98G-TMZ cells expressing GFP-NUPR1 was silenced. Co-IP analysis revealed that NUPR1 could bind to KDM3A, and this interaction was enhanced under hypoxia, which could be reduced by si-NUPR1 (Fig. 3B). Additionally, H3K9me2 expression pattern was markedly reduced in the hypoxia group relative to the normoxia group, while si-NUPR1 treatment could restore H3K9me2 expression in hypoxic TMZ-treated cells (*p* < 0.05, Fig. 3C). Collectively, our findings indicate that hypoxia could reduce H3K9me2 expression in glioma cells by promoting the binding of NURP1 to KDM3A.

### KDM3A overexpression partially reversed silencing NURP1-induced repression of glioma cell autophagy by limiting H3K9me2 levels

**Figure 4:**
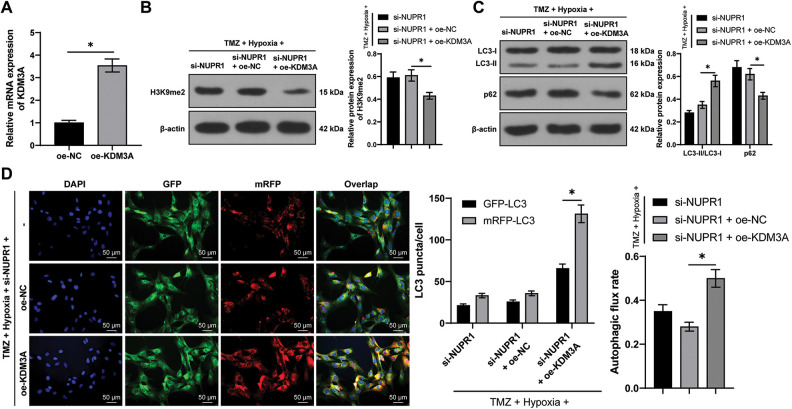
KDM3A overexpression partially reversed the repression of silencing NURP1 on glioma cell autophagy via limiting H3K9me2 levels. Plasmid oe-KDM3A was transfected to U251-TMZ cells. (A) qPCR was used to examine KDM3A expression. Hypoxia-treated U251-TMZ cells were transfected with si-NUPR1 and oe-KDM3A. (B) Western blot was conducted to examine H3K9me2 expression. (C) Western blot was performed to verify the expressions of LC3-II/LC3-I and p62; (D) mRFP-GFP-LC3 dual-fluorescence labeling was used to verify autophagy flux and calculate autophagy flux rate. The cell experiment was conducted 3 times independently; the data were expressed as mean ± standard deviation; data in panel A were verified using *t*-test; data in panels B and D (right) were analyzed using one-way ANOVA, followed by Tukey’s multiple comparison test; data in panels C and D (left) were analyzed using two-way ANOVA, followed by Tukey’s Honestly-Significant-Difference test; *p < 0.05.

U251-TMZ cells were transfected with oe-NC or oe-KDM3A to upregulate KDM3A expression. Relative to the oe-NC group, KDM3A expression was risen in the oe-KDM3A group (*p* < 0.05, Fig. 4A). Rescue experiments were then conducted in TMZ and si-NUPR1-treated hypoxic U251-TMZ cells. The H3K9me2 expression in the si-NUPR1 + oe-KDM3A group was lower relative to the si-NUPR1 + oe-NC group (*p* < 0.05, Fig. 4B). Additionally, relative to the si-NUPR1 + oe-NC group, the si-NUPR1 + oe-KDM3A group showed elevated the LC3-II/LC3-I expression and autophagic flux rate while p62 expression was reduced (*p* < 0.05, Figs. 4C and 4D), indicating autophagic flux was promoted. Collectively, the aforementioned findings suggest that KDM3A could partially reverse the repression of glioma cell autophagy initiated by NURP1 silencing by suppressing H3K9me2 levels.

### NURP1 binding to KDM3A limited H3K9me2 levels and further promoted TFEB transcription

**Figure 5:**
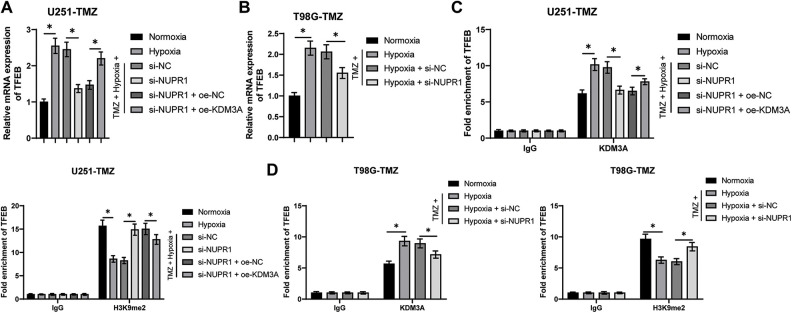
NURP1 binding to KDM3A limited H3K9me2 levels and further promoted TFEB transcription. Hypoxia-treated U251-TMZ cells were transfected with si-NUPR1 or/and oe-KDM3A. (A) qPCR was adopted to examine TFEB transcription level. Hypoxia-treated T98G-TMZ cells were transfected with si-NUPR1. (B) qPCR was adopted to examine TFEB transcription level. (C) Ch-IP was conducted to identify the enrichment of KDM3A and H3K9me2 in the TFEB promoter region in U251-TMZ cells; (D) Ch-IP assay was performed to identify the enrichment of KDM3A and H3K9me2 in the TFEB promoter region in T98G-TMZ cells. The cell experiment was conducted 3 times independently; the data were expressed as mean ± standard deviation; data in panels A and B were analyzed using one-way ANOVA, followed by Tukey’s multiple comparison test; data in panels C and D were analyzed using two-way ANOVA, followed by Tukey’s Honestly-Significant-Difference test; *p < 0.05.

Existing evidence suggested the involvement of TFEB in the biogenesis of lysosomes and the adjustment of autophagy by inducing the expressions of genes related to autophagy and lysosomes [33]. Relative to the normoxia group, the TFEB transcription level was higher in the hypoxia group, but si-NUPR1 decreased TFEB transcription (*p* < 0.05, Figs. 5A and 5B), while oe-KDM3A resulted in an increase in TFEB transcription (*p* < 0.05, Fig. 5A). Ch-IP assay revealed that KDM3A enrichment was increased in the hypoxia group relative to the normoxia group, while si-NUPR1 treatment decreased KDM3A enrichment, and H3K9me2 enrichment showed the opposite trend (*p* < 0.05, Figs. 5C and 5D). Additionally, oe-KDM3A could partially reverse the effects of si-NUPR1 (*p* < 0.05, Fig. 5C). These results suggest that silencing NURP1 may limit TFEB transcription by increasing H3K9me2 levels through reducing KDM3A enrichment in the TFEB promoter region.

### TFEB overexpression partially reversed silencing NURP1-induced repression of glioma cell autophagy

**Figure 6:**
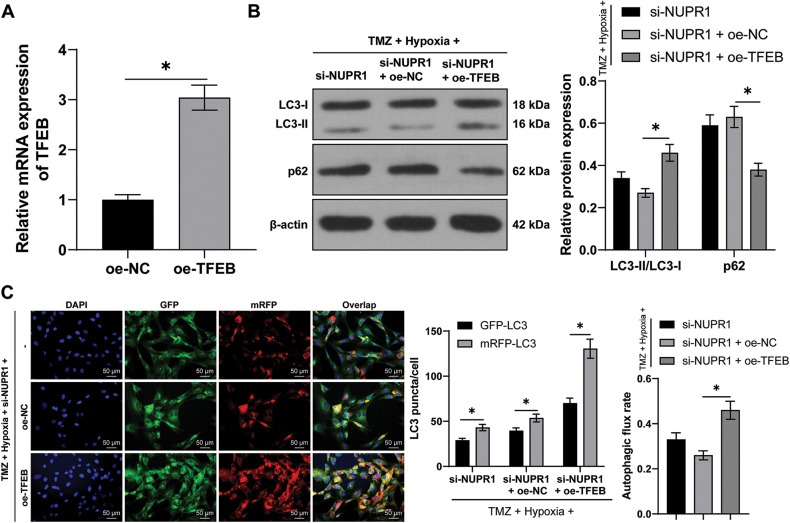
TFEB overexpression partially reversed the repression of silencing NURP1 on glioma cell autophagy. U251-TMZ cells were transfected with oe-TFEB. (A) qPCR was used to examine TFEB expression. Hypoxia-treated U251-TMZ cells were transfected with si-NUPR1 and oe-TFEB. (B) Western blot was used to examine the expressions of LC3-II/LC3-I and p62; (C) mRFP-GFP-LC3 dual-fluorescence labeling was performed to detect autophagy flux and calculate autophagy flux rate. The cell experiment was conducted 3 times independently; the data were expressed as mean ± standard deviation; data in panel A were verified using *t*-test; data in panel C (right) were analyzed using one-way ANOVA, followed by Tukey’s multiple comparison test; data in panels B and C (left) were analyzed using two-way ANOVA, followed by Tukey’s Honestly-Significant-Difference test; *p < 0.05.

The plasmid oe-TFEB was utilized to overexpress TFEB in U251-TMZ cells (*p* < 0.05, Fig. 6A). Next, the transfected U251-TMZ cells of the si-NUPR1+ hypoxia group underwent rescue experiments. Relative to the si-NUPR1 + oe-NC group, LC3-II/LC3-I expression and autophagic flux rate were elevated whereas the p62 expression was decreased in the si-NUPR1 + oe-TFEB group (*p* < 0.05, Fig. 6B). The aforementioned findings suggested that silencing NURP1 repressed cell autophagy by limiting TFEB expression.

### Silencing NUPR1 repressed cell autophagy to reduce TMZ resistance in glioma in vivo via the KDM3A/TFEB axis

**Figure 7:**
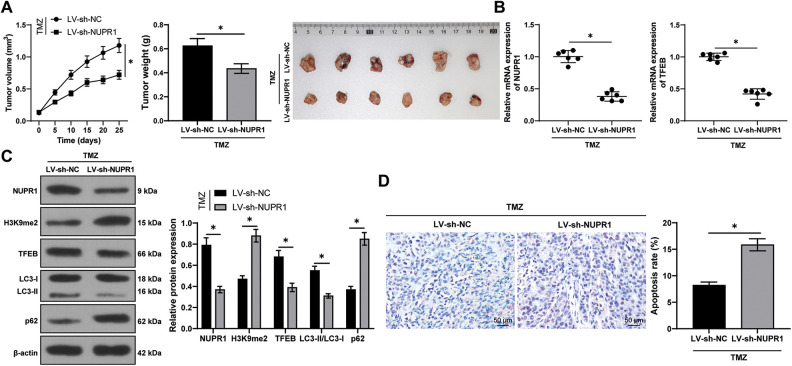
Silencing NUPR1 repressed cell autophagy to reduce TMZ resistance in glioma *in vivo* via the KDM3A/TFEB axis. (A) A xenograft tumor model was established to obtain the growth curve, the solid diagram, and the statistical result of the tumor weight; (B) qPCR was used to determine the expressions of NUPR1 and TFEB; (C) Western blot was performed to examine the expressions of NUPR1, H3K9me2, TFEB, LC3-II/LC3-I, and p62; (D) TUNEL staining was conducted to verify cell apoptosis in the tumor tissues. N = 6. The data were expressed as mean ± standard deviation; data in panels A (bar chart), B, and D were verified using *t*-test; data in panels A (line chart) and C were analyzed using two-way ANOVA, followed by Tukey’s Honestly-Significant-Difference test; *p < 0.05.

Finally, to validate the aforementioned mechanism *in vivo*, a subcutaneous xenograft tumor model was established in nude mice by injecting U251-TMZ cells followed by TMZ treatment. Relative to the LV-sh-NC group, the tumor growth rate and tumor volume were markedly decreased in the LV-sh-NUPR1 group (*p* < 0.05, Fig. 7A). Additionally, relative to the LV-sh-NC group, NUPR1 and TFEB expressions were decreased (*p* < 0.05, Figs. 7B and 7C), while LC3-II/LC3-I expression was reduced (*p* < 0.05, Fig. 7C) and H3K9me2 and p62 expressions were elevated in the LV-sh-NUPR1 group (*p* < 0.05, Fig. 7C). TUNEL staining revealed that the apoptotic rate was higher in the LV-sh-NUPR1 group than the LV-sh-NC group (*p* < 0.05, Fig. 7D). These findings suggest that silencing NUPR1 limits cell autophagy and reduces TMZ resistance in glioma *in vivo* through the KDM3A/TFEB axis.

## Discussion

Glioma represents the most common primary tumor among intracranial tumors, comprising of several types [34]. Temozolomide (TMZ) and radio-chemotherapy combination therapy have been shown to extend the survival time of patients with grade IV glioma, including GBM [35], gliomas often develop drug resistance to TMZ [36]. In this study, we evaluated the effects of nuclear protein-1 (NURP1) on TMZ resistance in hypoxia-treated glioma cells by modulating autophagy via the KDM3A/TFEB axis.

Glioma development is a complex process involving various genetic signals [37–39]. Notably, NURP1 has been demonstrated to confer resistance to multiple drugs in cancers, including tamoxifen, sorafenib, and docetaxel [13,15,40]. Given its involvement in drug resistance, we speculated that NURP1 may also contribute to TMZ resistance in glioma cells. To verify this hypothesis, TMZ-resistant cells U251-TMZ and T98G-TMZ were constructed and cultured under normoxia and hypoxia conditions. In consistency with previous studies, we found that hypoxia amplified TMZ resistance in glioma cells, as evidenced by the elevated IC_50_ values of TMZ and decreased apoptotic rate [41]. Moreover, NUPR1 expression was notably up-regulated in hypoxia-treated U251-TMZ and T98G-TMZ cells, thus eliciting a potential role of NURP1 in mediating TMZ resistance in hypoxia-treated glioma cells. To investigate the aforementioned speculation, we silenced NUPR1 expression in hypoxia-treated U251-TMZ and T98G-TMZ cells and observed a reduction in cell proliferation and an elevation in apoptosis. A relative study reported that silencing NUPR1 expression in GBM cells led to a decrease in GBM cell proliferation and migration [42]. The aforementioned findings indicated that silencing NUPR1 may contribute to TMZ resistance in hypoxia-treated glioma cells.

Furthermore, hypoxia has been found to induce and promote autophagy, affecting the progression of tumors and cancers. Reduced expression of autophagy marker proteins can inhibit glioma cell survival [10,43]. Therefore, we hypothesized that NUPR1 could maintain TMZ resistance in hypoxia-treated glioma cells by modulating autophagy. Silencing NUPR1 in hypoxia-treated U251-TMZ and T98G-TMZ cells led to a decreased in LC3-II/LC3-I expression and an increased p62 expression. However, rapamycin treatment resulted in opposite results. Additionally, silencing NUPR1 limited hypoxia-induced autophagy flux, while rapamycin promoted autophagy flux. To further verify that NUPR1 had an impact on TMZ resistance by modulating autophagy, we treated U251-TMZ and T98G-TMZ cells with TMZ at IC_50_ concentration. After rapamycin treatment, cell proliferation was enhanced, and apoptosis was suppressed. Existing studies have shown that NUPR1 suppression decreased autophagy and increased the apoptotic rate in multiple myeloma, but rapamycin reversed this increase [44]. Silencing NUPR1 hampered autophagic flux and promoted apoptosis, thereby increasing the sensitivity of hepatocellular carcinoma cell to sorafenib [45]. These findings suggest that silencing NUPR1 limited hypoxia-induced autophagy, and attenuates hypoxia-induced TMZ resistance. However, we did not probe the role of NUPR1 in the resistance of glioma cells to other chemotherapeutic drugs.

KDM3A is a specific demethylase for H3K9me2 and has been shown to be induced by hypoxia, promoting cancer cell invasion and contributing to radio-resistance [46,47]. Previous studies have reported an elevated KDM3A in myeloma cells in response to a decrease in H3K9me2 expression [48] and that KDM3A could stimulate autophagy by suppressing H3K9me2 levels [23]. In this study, we validated the binding of NUPR1 to KDM3A, which was enhanced by hypoxia and reduced by NUPR1 knockdown. A decreased H3K9me2 expression was observed after hypoxia treatment, but increased after silencing NUPR1. Furthermore, overexpression of KDM3A in U251-TMZ cells subjected to si-NUPR1 under hypoxia resulted in decreased H3K9me2 level and promoted autophagy. These findings are consistent with previous studies demonstrating that silencing H3K9me2 can accelerate autophagy activity in glioma cells [49], and that silencing KDM3A by EZH1 can inhibit M2 macrophage polarization and suppress GBM progression [22]. The aforementioned evidence indicated that KDM3A overexpression reverses the inhibitory effect of NURP1 knockdown on autophagy in glioma cell by limiting H3K9me2 levels. Additionally, previous studies have reported that KDM3A can be negatively regulated by miR-449a in lung cancer [50] and activated by the transcription factor of HIF-1α in multiple myeloma [51]. Therefore, miRNAs and transcription factors may be implicated in the upstream mechanism of KDM3A in glioma development.

For a comprehensive understanding of the effector of the KDM3A/NURP1 axis in glioma cell autophagy, it is vital to consider the role of TFEB in mediating the autophagy-lysosome system in various tumors [52]. TFEB activation induced autophagy and facilitated the progression of pancreatic cancer [53,54]. The suppression of TFEB-mediated autophagy increased apoptosis induced by salidroside in chondrosarcoma [55]. In our study, we observed that TFEB transcription level was elevated after hypoxia treatment and down-regulated by NUPR1, while overexpression of KDM3A led to increased TFEB transcription. Furthermore, hypoxia treatment increased the enrichment of KDM3A enrichment and decreased the enrichment of H3K9me2 in the TFEB promoter region, which was reversed by silencing NUPR1. Conversely, overexpression of KDM3A restored the roles of silencing NUPR1 in the enrichment of KDM3A and H3K9me2. These results illustrated that NURP1 binding to KDM3A regulated H3K9me2 and further facilitated TFEB transcription. To further assess the functionality of TFEB in glioma cell autophagy, U251-TMZ cells overexpressing TFEB were subjected to si-NUPR1 under hypoxia. Our findings demonstrated that overexpression of TFEB promoted autophagy. Consistently, in pancreatic ductal adenocarcinoma, TFEB suppression limited autophagy and attenuated gemcitabine resistance in pancreatic cancer cells [56]. Altogether, our findings suggest that TFEB overexpression partially restores glioma cell autophagy repressed by silencing NURP1.

Finally, we aimed to validate the aforementioned mechanism *in vivo* by establishing a mouse xenograft tumor model. Silencing NUPR1 led to slower tumor body and decreased tumor volume. Additionally, we observed that silencing NUPR1 reduced the TFEB expression, increased H3K9me2 level, limited autophagy, and increased the apoptotic rate *in vivo*. Collectively, these findings support the role of NUPR1 in promoting cell autophagy to reduce TMZ resistance in glioma *in vivo* via the KDM3A/TFEB axis. Nevertheless, the protein levels of TFEB were not determined. Additionally, whether NUPR1 could modulate other genes that participate in glioma cell autophagy and TMZ resistance under hypoxia remains unknown. In the future, we plan to employ genomics strategies, such as Ch-IP-Seq and microarrays, to identify additional targets of NUPR1 associated with autophagy and drug resistance in glioma cells.

**Figure 8:**
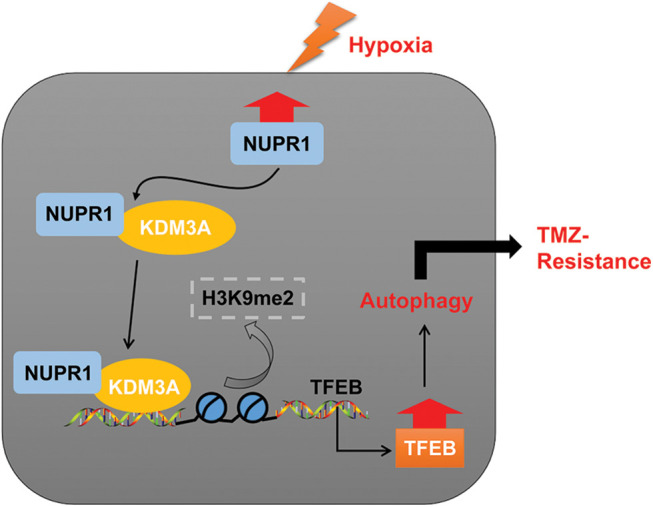
Mechanism diagram. NUPR1 promoted KDM3A enrichment in the TFEB promoter region via binding to KDM3A, and facilitated TFEB transcription via H3K9me2 demethylation, thereby accelerating autophagy under hypoxic conditions, and eventually enhancing TMZ resistance in glioma cells.

In essence, our experimental findings validated that NUPR1 binding to KDM3A reduces H3K9me2 levels and enhances TFEB transcription, leading to increased autophagy and augmenting TMZ resistance in hypoxia-treated glioma cells (Fig. 8). To the best of our knowledge, our study is the first to demonstrate the significance of the NUPR1/KDM3A/TFEB axis in TMZ resistance in glioma cells, and provides promising biomarkers for the treatment of glioma and a novel therapeutic approach to overcoming TMZ resistance.

## Data Availability

The data used to support the findings of this study are available from the corresponding author upon reasonable request.
